# Assessing airway inflammation in clinical practice – experience with spontaneous sputum analysis

**DOI:** 10.1186/1471-2466-8-5

**Published:** 2008-02-28

**Authors:** Olaf Holz, Tanja Seiler, Andreas Karmeier, Jan Fraedrich, Helmut Leiner, Helgo Magnussen, Rudolf A Jörres, Lutz Welker

**Affiliations:** 1 Hospital Großhansdorf, Center for Pneumology and Thoracic Surgery, 22927 Großhansdorf, Germany; 2 Outpatient clinic for internal medicine and pneumology, 20354 Hamburg, Germany; 3 Outpatient clinic for internal medicine and pneumology, 21029 Hamburg, Germany; 4 Outpatient clinic for internal medicine and pneumology, 24148 Kiel, Germany; 5 Institute and Outpatient Clinic for Occupational and Environmental Medicine, Ludwig-Maximilians-University, Munich, Germany

## Abstract

**Background:**

The assessment of airway inflammation for the diagnosis of asthma or COPD is still uncommon in pneumology-specialized general practices. In this respect, the measurement of exhaled nitric oxide (NO), as a fast and simple methodology, is increasingly used. The indirect assessment of airway inflammation, however, does have its limits and therefore there will always be a need for methods enabling a direct evaluation of airway inflammatory cell composition. Sampling of spontaneous sputum is a well-known, simple, economic and non-invasive method to derive a qualitative cytology of airway cells and here we aimed to assess today's value of spontaneous sputum cytology in clinical practice.

**Methods:**

Three pneumologists provided final diagnoses in 481 patients having sputum cytology and we retrospectively determined posterior versus prior probabilities of inflammatory airway disorders. Moreover, in a prospective part comprising 108 patients, pneumologists rated their confidence in a given diagnosis before and after knowing sputum cytology and rated its impact on the diagnostic process on an analogue scale.

**Results:**

Among the 481 patients, 45% were diagnosed as having asthma and/or airway hyperresponsiveness. If patients showed sputum eosinophilia, the prevalence of this diagnosis was elevated to 73% (n = 109, p < 0.001). The diagnosis of COPD increased from 40 to 66% in patients with neutrophilia (n = 29, p < 0.01).

Thirty-three of the 108 patients were excluded from the prospective part (26 insufficient samples, 7 incomplete questionnaires). In 48/75 cases the confidence into a diagnosis was raised after knowing sputum cytology, and in 15/75 cases the diagnosis was changed as cytology provided new clues.

**Conclusion:**

Our data suggest that spontaneous sputum cytology is capable of assisting in the diagnosis of inflammatory airway diseases in the outpatient setting. Despite the limitations by the semiquantitative assessment and lower sputum quality, the supportive power and the low economic effort needed can justify the use of this method, particularly if the diagnosis in question is thought to have an allergic background.

## Background

Airway inflammation is a hallmark of asthma [1] and chronic obstructive pulmonary disease (COPD) [2], and there is evidence that the assessment of inflammation is useful for diagnosis [3-5]. In addition, monitoring inflammation by induced sputum analysis or the measurement of exhaled nitric oxide (NO), led to a lower frequency of exacerbations in asthma [6,7], and allowed to reduce the dose of inhaled corticosteroids [8].

The induction and processing of sputum for a quantitative differential cell count is, however, very labour-intensive, time-consuming and therefore not suited to be performed in outpatient clinics. The analysis of NO is fast and simple and offers many of the ideal features a tool to assess asthma control should provide [9]. However, it requires the investment for an NO-analyser and, more importantly, provides only indirect evidence for eosinophilic airway inflammation, which can be difficult to interpret, when e.g. high NO values persist, despite increases in steroid dose [10]. In these cases, an additional direct assessment of the type of airway inflammation could be helpful.

The use of spontaneous sputum, on the other hand, is simple, as samples can be produced by the patients at home. Although today not routinely used, it has a long history as a tool in pneumology. It does not require investments for equipment and cytological analysis of smear slide preparations are generally reimbursed by health insurances (e.g. in Germany). However, samples are often of lower quality than induced sputum, partly due to the unsupervised production and the delayed analysis of mailed samples. In addition, smear slides samples provide only semiquantitative information. Despite these obstacles, it seems worthwhile to assess the diagnostic value of this economic methodology in pneumological practices.

We thus analysed the relationship between final diagnosis and cytological result for spontaneous sputum samples of 481 patients provided by pneumology-specialised general practitioners. In this retrospective part, we calculated the prior probability for different airway diseases as well as their posterior probabilities for patients with pronounced eosinophilic or neutrophilic inflammation. This approach provided the information that was used to estimate the diagnostic value of spontaneous sputum analysis. In a prospective part including 108 newly recruited patients without known diagnosis, three pneumologists were asked to rate their confidence in a specific diagnosis asserted before and after cytology, as well as the subjective importance of the cytological result for the respective diagnostic process.

## Methods

### Patients and pneumology-specialized general practitioners

The co-operating pneumology-specialized general practitioners of the Hamburg/Kiel area covered internal medicine with focus on pneumology. Each sputum sample was mailed to the cytologist and accompanied by information on the suspected indication (inflammatory disorder, suspected tumour, hemoptysis, dust exposure, unknown). Irrespective of this, the cytologist (L.W.) always put attention to all these aspects. Samples as well as patients' characteristics were coded and all other participating researchers were blinded with the respect to the identity of the patient.

### Spontaneous sputum samples

Patients received a 50 mL tube and a labelled envelope. They were instructed how to produce sputum in the morning at home and to immediately send the tube to the Cytological Laboratory of the Hospital Großhansdorf, where smear slides were prepared and stained by Giemsa within one day. The time delay between sputum production and processing ranged between 24 and 48 h. Slides were first rated for quality (sufficient, limited, not suited) and whether sufficient airway cell numbers were present. Slides were then scored semiquantitatively, as showing high, medium, or low numbers of inflammatory cells, with emphasis on eosinophils and neutrophils. The terms eosinophilia and neutrophilia refer to slides with high counts of these cells. In addition, the presence of tumour cells or other suspicious alterations was recorded. The pneumologist was informed about the result within one day after analysis.

### Retrospective part

Data from samples collected over a 2-year period were included. To reduce the variability arising from differences between the pneumologists' habits, only the three physicians with the largest numbers of patients were included. Final diagnoses were extracted from 481 patient files of these physicians. The diagnosis of asthma and COPD was based on international guidelines [1,2]. To accommodate for remaining differences in terminology, final diagnoses were grouped into seven major categories: (1) healthy, (2) asthma and/or bronchial hyperresponsiveness (BHR), (3) COPD/bronchitis, (4) pneumonia, (5) alveolar haemorrhage, (6) tumour, (7) others. Frequencies according to the final diagnosis were taken as prior probabilities for each diagnostic group. Sputum samples were categorised into the same groups, taking into account the whole pattern of alterations. Especially eosinophils and neutrophils allowed to state cytological diagnoses for groups (2) and (3). The comparison between final diagnosis and the specific cytological findings or the cytological diagnostic category then allowed to compute posterior probabilities. This type of analysis was restricted to groups (2) and (3), as these comprised the majority of cases. The change from prior to posterior probability was taken as a measure of the predictive value of sputum analysis. We preferred to analyse the data this way, as it takes into account disease prevalence which is important under practical aspects.

### Prospective part

The same three pneumologists, blinded to the results of the retrospective analysis, were asked to include patients in whom the diagnosis of airway disease had not yet been established. Samples were collected over a 6-month period. Patients underwent the diagnostic procedures commonly used in the respective outpatient clinic. The pneumologists were then asked to rate their confidence in the diagnosis without knowing sputum cytology (Rating 1). Within 5 days after having received the cytological result, the pneumologists then repeated the rating (Rating 2). Each rating was performed on a separate analogue scale for each of the 7 diagnostic groups asthma, COPD, infection/pneumonia, BHR, tuberculosis, reflux, tumour. Scales were labelled "unsure" (0 mm) and "sure" (100 mm) and the rating was quantified in mm. The results of the repeated ratings were compared (changes from Rating 1 to Rating 2) and grouped into four classes: (C) increase in distance = diagnosis confirmed by sputum and confidence in the diagnosis increased, (N) change in diagnostic group = sputum providing new clues for diagnosis, (U) distance unchanged = no information, (L) reduction in distance = sputum rendering diagnosis less likely but not providing new clues.

In addition the pneumologists were asked to answer the following questions on analogue scales (Rating 3) labelled "do not agree" (0 mm) and "agree" (100 mm): knowledge of sputum cytology either confirmed the diagnosis, or had an impact on treatment, or enabled a faster final diagnosis, or had saved costs, or resulted in a more successful treatment, or provided new diagnostic and/or therapeutic clues.

### Statistical analysis

Prior and posterior probabilities were compared with each other by the χ^2^-statistics. Median values and quartiles of distances on analogue scales (mm) were calculated. The Wilcoxon matched-pairs signed-ranks test was used for the analysis of changes of analogue scales. Statistical significance was assumed for p < 0.05.

## Results

### Retrospective part

Within 2 years 1434 samples from 1147 patients were sent to the Cytological Laboratory by 25 pneumology-specialized general practitioners. In 63% of cases the indication to ask for sputum analysis was a suspected inflammatory disorder, followed by 18% with suspected tumour, 8% with suspected haemoptysis, and 11% without specified indication. Samples were of sufficient quality in 78% of patients and of limited quality in 22%, mainly due to the presence of large proportions of saliva. In the subgroup of 481 patients with known final diagnosis the pneumologists suspected an inflammatory disorder in 79% of cases, followed by 8% with suspected tumour. Sputum quality in this subset was similar to that of the total group (77% sufficient, 23% limited). In case of repeated determinations the first adequate sample of a patient was used for the analysis.

Table 1 lists the relative frequencies of diagnoses for all sputum samples (prior probabilities), as well as for the subgroups showing high eosinophil counts and high neutrophil counts (posterior probabilities). If there was marked eosinophilia irrespective of neutrophil counts (n = 109), the probability of the asthma/BHR final diagnosis was raised from 45 to 73% (χ^2^: p < 0.0001). Conversely, if neutrophilia was pronounced (n = 29), that of COPD/bronchitis was increased from 40 to 66 % (χ^2^: p = 0.008). Three patients showed a final diagnosis of bronchitis/COPD and both marked eosinophilia and marked neutrophilia. A neutrophilic response can also be expected in pneumonia. Despite a low a priori probability (3%) we also observed a significant increase to 13% in samples with high neutrophil counts (χ^2^: p = 0.05). When considering the 369 sputum samples of sufficient quality only, similar results were obtained (p = 0.002, 0.001 and 0.01, respectively). When considering only those patients, whose sputum was submitted to evaluate a potential inflammatory disorder (n = 382), results were similar (p = 0.007, 0.006 and 0.03, respectively).

**Table 1:** Results of retrospective analysis of spontaneous sputum samples

		Prior Probability (in %)	Posterior probability after sputum analysis (in %)	
Final diagnosis	n		Eosinophilia	Neutrophilia
Healthy	3	0.6	0.0	0.0
Asthma/BHR	216	44.9	72.5	13.8
COPD/bronchitis	194	40.3	22.9	65.5
Pneumonia	16	3.3	0.0	13.8
Alveolar haemorrhage	4	0.8	0.0	0.0
Tumour suspicious	19	4.0	2.8	6.9
Others	29	6.0	1.8	0.0
Total	481	100	100	100
n corresponding to column		481	109	29

The table presents the percentages of patients according to final diagnosis (prior probabilities) as well as posterior probabilities according to the two major outcomes (eosinophilia and neutrophilia) of the semiquantitative sputum analysis. Only these two diagnostic categorie are shown, as numbers regarding other sputum findings were too low for statistical analysis.

In some of the 481 samples, other cytological aspects such as the degree of degranulation, number of siderophages, Charcot-Leiden crystals or pigment within cells were noted. The frequency of these features was too low for quantitative analysis, although they might have had an impact in individual samples.

### Prospective part

**Figure 1:**
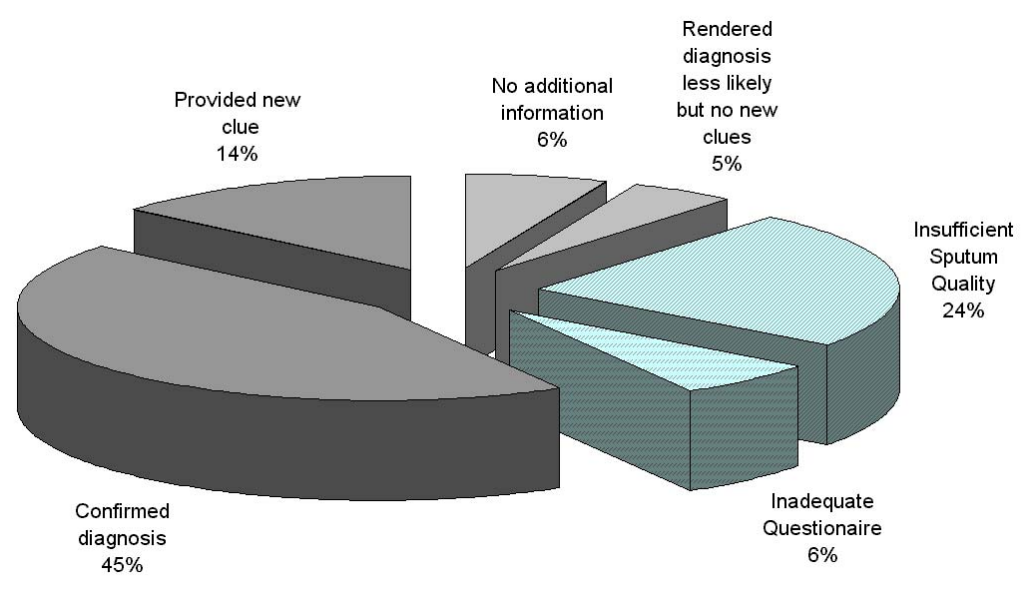
**The impact of spontaneous sputum cytology on the diagnostic process of 108 new patients of 3 pneumology-specialized general practitioners.** Patients underwent the diagnostic procedures commonly used in the respective practice. The pneumologists then rated their confidence in the diagnosis before knowing sputum cytology and again after having received the cytological result. The repeated ratings on analog scales were compared: increase in distance = diagnosis confirmed, change in diagnostic group = sputum provided new clues, distance unchanged = no additional information, reduction in distance = sputum rendered diagnosis less likely but provided no new clues.

A total of 108 patients were included into this analysis, of whom 26 (24%) did not produce adequate sputum samples. These patients as well as further 7 patients with incomplete questionnaire data were omitted. The remaining 75 patients were categorised according to the diagnostic scores and questionnaire data provided by the pneumologists (Figure 1, Table 2).

**Table 2:** Results of prospective analysis of spontaneous sputum samples.

Question		Diagnosis confirmed	Analysis had impact	Diagnosis faster	Diagn. more economic	Success of treatment	New clue for diagn.
Category according to change in analog scales of diagnoses	n	mm	mm	mm	mm	mm	mm
Overall	75	**80 (52;94)**	**85 (50;92)**	**85 (52;95)**	50 (45;85)	55 (50;87)	10 (2;50)
C (confirmed, more confident)	48	**90 (80;100)**	**90 (65;97)**	**90 (85;100)**	50 (50;90)	**80 (50;90)**	10 (3;15)
N (new clue)	15	50 (15;60)	**80 (65;90)**	60 (50;85)	50 (50;60)	60 (50;87)	**86 (50;90)**
U (unchanged)	7	52 (5;55)	45 (5;50)	50 (5;52)	42 (5;50)	45 (40;50)	25 (5;38)
L (less likely, no new clues)	5	75 (15;100)	50 (15;80)	70 (15;100)	15 (12;50)	50 (15;50)	15 (12;50)

Data represent median (quartiles) distances in mm on the analog scales of Rating 3 assessed after sputum analysis, whereby 0 indicates a negative and 100 a positive (affirmative) answer. To check the association of these general ratings with the changes in the rating regarding specific diagnoses, answers were further grouped (C, N, U, L) according to the comparison between Rating 1 (prior to sputum analysis) and Rating 2 (performed after having received the sputum result). The bold face type indicates the scores ≥ 80 in Rating 3.

Within category "C" (suspected diagnosis confirmed, more confident) sputum eosinophilia played the major role (30/48 cases). Correspondingly, the "confirmed"-rating for asthma and/or BHR (n = 29) and/or rhinitis (n = 1) increased from 71(50;87) to 91(75;95) mm (median (quartiles)) (Wilcoxon: p < 0.001). In 9 patients a marked neutrophilia was considered as confirming the initial diagnosis of COPD, infection or pneumonia and in one patient neoplastic changes confirmed the suspected tumour diagnosis.

Regarding category "N" (new clue), the presence or absence of eosinophilia was responsible for a change in the likelihood of the diagnosis in 12 patients. In the remaining 3 patients non-specific inflammation resulted in increases for the confidence into a COPD or infection or pneumonia diagnosis. For both categories "C" and "N", pneumologists reported that the sputum analysis had influenced the further treatment of the patient, by giving a high score in Rating 3 ("impact").

In category "U" the low scores in the questionnaire indicated that the pneumologists did not find sputum cytology particularly helpful in these instances, either due to an already well-established diagnosis, or due to inconspicuous findings of weak non-specific inflammation.

In category "L" sputum cytology led to a decrease in the score of the suspected diagnosis, without providing new clues, in 5 patients. For both categories "U" and "L", variability within analogue scales was large, indicating that the information by sputum cytology was not considered generally informative.

## Discussion

The results of this study indicate that spontaneous sputum cytology is feasible and can assist in the diagnostic process in pneumology-specialized general practices. As expected, spontaneous sputum eosinophilia was associated with an increased likelihood of asthma or related syndromes. Although sputum production at home resulted in about one quarter of inadequate samples, about 80 % of the remaining cases showed a diagnostic impact as reported by the pneumologists. Provided that the limitations of the semiquantitative method are acknowledged, the use of spontaneous sputum therefore offers pneumology-specialized general outpatient clinics a simple and economic way to support the diagnosis of airway diseases by yielding clues on airway inflammation. In this respect, it could also assist in cases, where exhaled NO measurements do not provide conclusive data.

The study was designed to assess the usefulness of spontaneous sputum under the same conditions as encountered in daily clinical practice. We thus tried to obtain data without interfering with the routine procedures followed by each of the participating pneumologists. Despite adherence to national and international guidelines, each practitioner followed a diagnostic process that took the needs of individual patients as well as the own experience into account. This also implied that we did not prescribe the set of allowed diagnoses which, as a consequence, comprised syndromes such as BHR in addition to asthma. Even in the prospective part we completely left the preferred approach of diagnosing and the sequence of measurements to the pneumologists and did not ask to follow a specific protocol, as commonly used e.g. in multicenter clinical trials.

The decision on requiring sputum cytology was also left to the pneumologists. It was based on the patient's clinical history and the results of standard diagnostic procedures (patients' history, physical examination, symptoms, measurement of lung function, reversibility, and hyperresponsiveness). The pneumologists, all of them with specific experience over ≥10 years, founded their initial diagnosis on these results. As the prospective part of the study indicated, the pneumologists already expressed a high confidence into their initial diagnosis in most patients and often asked for sputum cytology for confirmation. Accordingly, the ratings generally increased after knowing the sputum result. Taken together with positive scores in the first three questions of the questionnaire ("confirm", "impact", "faster"), this suggests that sputum cytology was considered as helpful. In addition, adequate treatment was felt to be established with more confidence and in shorter time as compared to cases without sputum data. This also might explain why sputum analysis has been used regularly over time by many pneumologists in our area.

In most instances, in which cytology provided new clues, the finding of eosinophilia redirected the pneumologist's attention towards an allergic disease such as asthma. Although new clues were found in only 10% of all cases, this seems to be an important observation, demonstrating that even limited additional information on airway inflammation as supplied by a semiquantitative method has the power to alter the opinion of an experienced physician. At the time of the study, measurements of NO were not available in clinical practices. It might well be that many patients with eosinophilia would also have been detected using NO [3,11]. We do not believe, however, that this renders spontaneous sputum analysis useless, as NO is known to provide very limited information in patients with neutrophilic inflammation, as found in COPD, current smokers, infections, or pneumonia [3].

We are well aware of the fact that the freedom in choosing diagnostic procedures introduced additional variability and potentially also a bias. The information policy in the prospective part of the study, however, prevented the pneumologists from using sputum data ahead of time, and the adherence to usual clinical habits ensured that the results reflected the situation of clinical practice. The study was performed in collaboration with pneumologists who had long experience in the use of spontaneous sputum. This might have favoured the inclusion of patients in whom sputum cytology tended to be particularly successful. In fact, the focus was on inflammatory processes, adding up to 79% of all submitted samples.

In the retrospective analysis we observed that the presence of marked eosinophilia and neutrophilia beared significant information, by altering the posterior probability for asthma/BHR or COPD/bronchitis. Interestingly, there was a high number of patients with eosinophilia and the final diagnosis of COPD without asthma. This also occurred in the prospective part; in the patients in whom eosinophilia was stated (n = 4) it did, however, not alter the confidence in the initial diagnosis of either COPD, bronchitis, or infection. Possibly, patients had eosinophilic bronchitis, or showed exacerbations of COPD, as characterised by eosinophilia and an increase in BHR [12]. Being aware that eosinophilic airway inflammation is responsive to treatment with corticosteroids, the information of ongoing eosinophilic inflammation is important and able to assist in the decision on the quality of treatment irrespective of the diagnostic label of the disease.

The study was based on the experience gained in the analysis of approximately 5000 sputum samples in the past 10 years. It suggests that an experienced observer can score degree and type of inflammation in most samples, even without fixation and with a delay of 1–2 days prior to processing. Eosinophils are rather resistant to degradation and were often found with intact cytoplasm and granular staining. Neutrophils were more often destroyed and remained in groups. Due to their sensitivity to destruction and the time delay it might be that neutrophilic inflammation has been underestimated in the samples. Therefore spontaneous sputum is more likely to display eosinophilic inflammation and consequently to help especially in the diagnosis of asthma, allergic airway diseases [13,14], as well as steroid responsive airway diseases. These factors can also be addressed indirectly by the measurement of exhaled NO, however currently no studies are available comparing the clinical usefulness and reliability of these different ways of analysing airway inflammation.

## Conclusion

In summary the results of this study showed that spontaneous sputum cytology can be helpful in the diagnosis of inflammatory airway diseases. Although it provided only semiquantitative and delayed information, it had the advantage to be feasible and not to require investments of time and money. Thus spontaneous sputum analysis offers a way for pneumologists to assess airway inflammation within outpatient settings. The same is probably true for GPs with focus on internal medicine. If NO measurement is available, sputum analysis probably still has the potential to assist in cases where NO does not lead to useful results. Our observations favour an increased use of spontaneous sputum analysis, especially in the assessment of suspected diagnoses of allergic background.

## Abbreviations

COPD : chronic obstructive pulmonary disease. NO: nitric oxide. BHR: bronchial hyperresponsiveness.

## Competing interests

The author(s) declare that they have no competing interests.

## Authors' contributions

OH, RJ and LW conceived the study, the design, performed the data analysis and statistics and drafted the manuscript. LW performed the sputum analysis. TS collected the data, participated in drafting the manuscript and coordinated the study. AK, JF, HL, and HM helped in data collection and critically revised the manuscript. HM secured the funding of the study. All authors read and approved the final manuscript.

## Pre-publication history

The pre-publication history for this paper can be accessed here:


